# Beyond BCMA, why GPRC5D could be the right way: treatment strategies with immunotherapy at relapse after anti-BCMA agents

**DOI:** 10.1007/s00262-023-03559-4

**Published:** 2023-11-04

**Authors:** Maria Livia Del Giudice, Sara Galimberti, Gabriele Buda

**Affiliations:** Department of Clinical and Experimental Medicine, Hematology, University of Pisa, 56126 Pisa, Italy

**Keywords:** Multiple myeloma, BCMA, GPRC5D, Talquetamab, Car-t, Therapy sequencing

## Abstract

Multiple Myeloma remains incurable, and there is a need for therapies with novel mechanisms of action. Recently, B cell maturation antigen targeted therapy has demonstrated deep and durable responses in a largely treated population. However, the relapse rate of myeloma patients after anti-BCMA treatment strategies is increasing worldwide, and one of the most challenging issues for them is to choose the best therapy sequencing. After anti-BCMA treatment, retreatment with anti-BCMA drugs remains an option, but new targets are emerging strongly. One of them is G protein-coupled receptor, class C group 5 member D (GPRC5D), that due to the very promising data from the use of chimeric antigen receptor T-cells (CAR-T) and bispecific antibodies (BsAb) seems to be the ideal candidate in the relay of myeloma treatment at relapse. In this literature review, we discuss data from treatment with the new drugs at relapse after anti-BCMA therapies, observing an undeniable benefit from the use of drugs directed against GPRC5D.

**Supplementary Information:**

The online version contains supplementary material available at 10.1007/s00262-023-03559-4.

## Introduction

Multiple myeloma (MM) is a tumor of the plasma cells. Its clinical course is characterized by relapses over time, with the progression-free interval decreasing with each relapse. More than 12,000 myeloma-related deaths are expected in 2023 in the USA, and despite increasing survival rates worldwide [1], the disease remains incurable. In particular, the estimated survival of a myeloma patient is dramatically reduced after the use of the major available drugs [2, 3]. Only recently, however, therapies directed against B cell maturation antigen (BCMA) have been approved and they have yielded unhoped-for results in triple-class exposed patients, who otherwise would have had very poor overall survival.

B cell maturation antigen, also known as TNFRSF17 or CD269, is a member of the tumor necrosis factor receptor family [4]. BCMA gene is located on chromosome 16 (16p13). It was first characterized from human malignant T-cell lymphoma cells; it was later shown that BCMA gene is preferentially expressed in mature B cells, suggesting a role for this gene in the B-cell developmental process [5, 6]. BCMA is indeed highly expressed by mature B cells, with upregulation in the late stages of normal B-cell differentiation and also on MM plasma cells [7, 8]; it is also essential for the survival of long-lived bone marrow plasma cells [9].

Ligands for BCMA include B-cell activating factor (BAFF) and a proliferation-inducing ligand (APRIL), and they act as growth and proliferation signals, attenuating cell death mechanism associated with NF-κB activation and playing as a survival factor, with maintenance and survival of malignant MM cells [10, 11]: this is probably why therapeutic strategies for blocking the BCMA pathway have proven to be so effective in MM. Recently, there has been a wide availability of different drugs and classes against BCMA: antibody–drug conjugates (ADC), bispecific antibodies (BsAb), chimeric antigen receptor T-cells (CAR-T) products, mainly used in randomized trials and also in clinical practice in advanced stages of MM, following the most common drugs, such as proteasome inhibitors (PI), immunomodulatory drugs (IMiD), anti-CD38 and anti-signaling lymphocyte activation molecule F7 (SLAMF7) monoclonal antibodies (mAb).

The use of therapies directed against BCMA has shown revolutionary results in heavily pretreated relapse and/or refractory multiple myeloma (RRMM) patients, as emerging from retrospective observations and indirect comparisons [12–14], with a great survival benefit compared to the anti-BCMA naive population [12], with results never achieved before especially with the use of CAR-T products, as shown extrapolating data from landmark studies about CAR-T targeting BCMA, Cartitude1 and KArMMA1, and the expected outcomes of the real-world population with RRMM with similar overall characteristics to the one enrolled in these trials [13, 14].

However, despite these enormous achievements, even such therapies are destined to exhaust the ability to control the disease and worldwide there is an increasing population of patients already exposed to anti-BCMA agents and already relapsed.

The mechanisms leading to relapse after anti-BCMA therapy are not fully understood.

Although BCMA is considered essential for the survival of bone marrow plasma cells [9], they may include antigen loss, considered a rare event, due to biallelic deletion of the BCMA locus on chromosome 16 [15–17] or reversible downregulation of BCMA expression following anti-BCMA therapies [18], eliminating the presence of BCMA on the extracellular surface and preventing the binding to targeted therapies. In other cases, as recently demonstrated, they may include antigen escape phenomena [19], in which mutational events occur in the extracellular domain of TNFRSF17 on functional BCMA epitopes, maintaining detectable BCMA expression on the surface, but inhibiting BCMA binding activity to targeted therapies and abrogating their cytolytic activity. These mutations do not appear to affect signaling resulting from APRIL ligand binding and downstream NF-κB activation. Other mechanisms of relapse may include the action of plasma cell membrane-mediated *γ*-secretase shedding, resulting in the formation of circulating soluble BCMA (sBCMA) [20] with the reduction in functional antigen to therapeutic binding along with the formation of high levels of sBCMA, potentially interfering with anti-BCMA therapies [21]. The development of anti-drug antibodies or T-cell depletion is also possible [22, 23], as is the emergence of a permissive microenvironment and the selection of subclones.

In relapse after a previous BCMA-targeting therapy, the persistence of the drug-binding site on plasma cells appears to provide an opportunity to re-use BCMA as a target in a sequential regimen [24].

However, there is no unanimous consensus on this issue, not least because even in the event of a response with such therapies, this is always temporary and destined to result in inevitable progression.

This is why it is time for treatments with new mechanisms of action, that can circumvent possible and even unknown mechanisms of acquired drug resistance.

G protein-coupled receptor, class C group 5 member D (GPRC5D), is a promising immunotherapy target that may provide a compelling offering in the treatment sequence after anti-BCMA therapies.

GPRC5D is an orphan receptor, coupled with a G-protein, with homology to members of fifth group of family C (associated with metabotropic glutamate receptor-like) of superfamily of mammalian G-protein coupled receptors (GPCRs), containing short amino terminal domain (ATD) of some 30–50 amino acids, in striking contrast all other family C members, that have very long ATD, of about 500–600 amino acids [25]. Its gene is located on chromosome 12 (12p13) [26]. Like many recently identified antigens, it has no known ligands, so its overall biological significance remains to be elucidated. It was originally described at two sites: the hair follicle [27–29] and the bone marrow of MM patients [30, 31]. Higher concentrations of this molecule have previously been shown to be associated with poor prognosis in multiple myeloma patients [30]. It has been described on the surface of malignant plasma cells using sensitive analysis techniques [32, 33]. In favor of its use as a clinical therapeutic target, GPRC5D has many advantages: it is almost exclusively restricted to cancer cells, with only low expression detected in normal tissues [30], it has BCMA-independent expression on plasma cells, which ensures persistence of expression even in relapse after therapy against BCMA [32], and it has shown excellent preclinical results in vivo in terms of efficacy and safety, independent of prior BCMA-targeted therapy and also including activity in a BCMA antigen escape model [33]. In addition, unlike other antigens targeted by MM therapies (including BCMA), there is no evidence that GPRC5D is released in peripheral blood.

## Relapse after BCMA-directed therapy: anti-BCMA retreatment option

When a patient with multiple myeloma relapses after direct therapy against BCMA, therapeutic sequencing is very challenging. Indeed, especially in BCMA persistence relapses, it is speculated that an alternative anti-BCMA agent may be used. However, the assessment of BCMA persistence is not a routinely performed practice and the escape model of relapse/resistance appears to be neither the only nor the main mechanism leading to relapse after such therapies [9, 15–23].

In addition, few data exist on this topic in clinical studies.

For example, the phase 2 DREAMM-2 trial, which led to the approval and reimbursement of belantamab mafodotin, first BCMA-targeted ADC, did not include patients who had previously received anti-BCMA therapies [34].

However, experience with the use of ADC after previous anti-BCMA therapies have inevitably accumulated, albeit from retrospective evidence and sub-analyses.

For example, the use of belantamab mafodotin after anti-BCMA therapies shows few inconsistent data, as shown in Table 1. However, with the exception of anecdotal cases, overall, this approach has shown limited efficacy [35–41].

**Table 1:** Belantamab mafodotin after anti-BCMA therapy

References	Study design	No. pts	Previous BCMA-Therapy	Response no. (QoR)
Vaxaman [35]	Retrospective analysis	7	Anti-BCMA CAR-T	0
Becmel [36]	Retrospective analysis	8	Anti-BCMA therapy nos	1 (PR); 1 (MR)
Van Oekelen [37]	Retrospective analysis	1	Anti-BCMA CAR-T	0
Reyes [38]	Retrospective analysis	7	Anti-BCMA CAR-T	2 (nos)
Rodriguez-Otero [39]	Retrospective analysis	10	Anti-BCMA CAR-T	ND; mPFS 15.5 mo
Gazeau [40]	Case reports	2	Anti-BCMA CAR-T	1 (VGPR); 1 (CR)
Cohen [41]	Case report	1	Anti-BCMA CAR-T	1 (MR)

*BCMA* B cell maturation antigen; *CR* complete response; *no.* number; *nos* not otherwise specified; *MR* minimal response; *PR* partial response; *QoR* quality of response; *VGPR* very good partial response

On the other hand, preliminary experience with BsAb after BCMA targeting therapies seem to do somewhat better. For example, ORR in patients already exposed to ADC or CAR-T against BCMA and treated with Teclistamab ORR was 38% and 45%, respectively, as reported from MajesTEC-1 cohort C, which enrolled 38 patients who had prior exposure to anti-BCMA treatment, but the general population from the trial reached an ORR of 62% [42]

In contrast, in the MagnetisMM-1 study evaluating the efficacy and safety of Elranatamab, another anti-BCMA BsAb, 22% of the enrolled population had already been exposed to BCMA-targeted therapy, with an ORR of 54% (7/13 patients) in the prior BCMA-treated patient population (lower than the general population, in which ORR was 64%) [43, 44], as summarized in Table 2. The results from the cohort B of MagnetisMM-3 (ClinicalTrials.gov ID NCT04649359), which will focus mainly on the already treated with anti-BCMA population, are not yet available.

**Table 2:** Anti-BCMA bispecific antibodies after BCMA targeting therapies

Investigational agent (study)	ADC exposed pts % (no.)	CAR-T exposed pts % (no.)	ORR (no.)	CRR or better (no.)	ORR in general population
Teclistamab (MajesTEC1) [42]	64 (16)	44 (11)	40 (10)	20 (5)	62
Elranatamab (MagnetisMM-1) [43–44]	14.4 (8)	16.4 (9)	54 (7)	23 (3)	64

*ADC* Antibody–drug conjugate; *BCMA* B cell maturation antigen; *CAR-T* chimeric antigen receptor T cell therapy; *CRR* complete response rate; *no.* number; *ORR* overall response rate; *pts* patients

In contrast, as far as CAR-T therapy is concerned, the outcome of patients already exposed to direct anti-BCMA therapy is a matter of great debate. Neither KArMMA1 nor CARTITUDE-1 enrolled this category of patients [45, 46], and this is why data focusing on this subject are of great interest [47–50].

Apart from anecdotal experience of good outcome with CAR-T after anti-BCMA ADC [41], the data from real-world experience are more numerically consistent. They show that, in particular, the use of Idecabtagene vicleucel (ide-cel) after prior anti-BCMA therapy is associated with inferior PFS and a lower likelihood of achieving both an overall response and a high-quality response [47, 49]. Ide-cel also describes a trend toward worse efficacy outcomes in patients who received it less than 6 months after their prior BCMA-targeted therapy (at more than 6 months after discontinuation, ORR is 83%; at less than 6 months, ORR is 60%). The timing of ide-cel infusion after prior BCMA-targeted therapy warrants further investigation [47], but an alert emerges in the early use of BCMA-directed CAR-T products immediately after relapse from drugs with the same target.

CARTITUDE-2, cohort C, is a phase 2 study evaluating safety and efficacy of Ciltacabtagene autoleucel (cilta-cel) after previous noncellular anti-BCMA immunotherapy, with a population divided as follows: 7 patients exposed to BsAb, 13 to ADC, and 1 of them exposed to both anti-BCMA exposed patients, obtaining response rate of 60%, which was lower than that of CARTITUDE-1 overall patients [46, 50]. It is also possible to find data on the sequencing of CAR-T products after relapse to first CAR-T treatment: after BCMA CAR-T relapse, patients may also respond to therapies to which they were previously considered refractory, but the duration of responses seems limited [38].

Taken together, what we observe at relapse after anti-BCMA treatment treated with same-targeted direct therapy is that, although this is a viable option, the expected outcomes are lower than in the anti-BCMA drug-naive population.

Currently, it is not yet possible to define the ideal therapeutic sequencing as the data we have analyzed are mainly from non-randomized studies.

In the absence of randomized comparisons, change of therapy could be a successful strategy and targeted therapy against GPRC5D seems to be very promising in the scenario of RRMM after anti-BCMA therapy.

## GPRC5D targeting therapies

GPR5C5D is currently the focus of thriving clinical and pre-clinical trial studies. Three CAR-T products directed against GPRC5D are today under development: MCARH109 [51], CC-95266 (BMS- 986,393) [52], OriCAR-017 [53].

These showed very good overall response rates (ORR) in heavily pretreated patients: respectively, ORR 71%, with complete response (CR) or better in 35% of patients, mDOR 7.8 months [51]; ORR 89%, ≥ CR in 47%, mDOR NR [52]; ORR 100%, ≥ CR in 60%, mDOR NR [53]. These results are very encouraging, but a longer follow-up is needed to assess the duration of the response over time.

In addition to cytokine release syndrome (CRS) and neurotoxicity, these CAR-T constructs have revealed new types of toxicity, not previously investigated: dysgeusia and skin and nail toxicity, most likely due to the sharing of the therapeutic target by different tissues and differences in pharmacological biodistribution compared to the past.

One of the CAR-T products, MCARH109 [51], also showed cerebellar toxicity, which does not appear to be seen in the other products in the same category.

Other drugs under development targeting GPRC5D are bispecific antibodies (BsAb). Several studies are underway testing T-cell redirecting GPRC5D bispecific antibodies in patients with relapsed and/or refractory MM, and they appear to be highly active in relapsed refractory patients. Talquetamab is an anti-GPRC5D bispecific antibody that has received breakthrough therapy designation from the US Food and Drug Administration (FDA) based on the data from the phase 1 MonumenTAL-1 trial [54]. Talquetamab showed favorable responses as a monotherapy in highly pretreated patients (median of 6 previous lines of therapy, with a range, 2 to 20) across different cohorts. CRS, skin-related events, and dysgeusia were common. Forimtamig, also known as RG6234, is another CD3 × GPRC5D bispecific antibody with a different configuration, having two GPRC5D binding sites (2:1 configuration). Early data suggest that RG6234 has similar response rates to talquetamab, although any impact of the 2:1 structure on efficacy needs to be evaluated using longer-term data [55].

Although utilization data can still be considered preliminary, we do know that studies evaluating the use of CAR-T versus GPRC5D constructs have included a large proportion of the population already exposed to BCMA-targeted therapy and have reported optimal results in this setting, as summarized in Table 3 [51–53]. The ORR results did not deviate from the general population, indicating that BCMA relapse does not affect the activity of a GPRC5D-targeted CAR-T product.

The GPRC5D-targeted BsAb trials also included a significant population of patients who had already received BCMA-targeted therapy: in MonumenTAL-1 almost 30% of RRMM had received BCMA-targeted therapy, with an ORR overlapping with the general cohort [54, 56]; for Forimtamig, about 20% of the population had received prior anti-BCMA therapies with a good response rate of about 56% [55]; in these cases, however, these are small populations that deserve further investigation, but are already particularly encouraging.

In summary, the sequencing of different immunotherapies remains a challenge: the mechanisms of resistance and relapse are still not fully understood. In addition, relapse after BCMA—targeted therapy is an emerging unmet medical need.

Although only from preliminary data, retrospective experience, and a few clinical trials, we know that despite some sporadic encouraging results, the efficacy outcomes of retreatment with anti-BCMA therapies appear to be lower than in the BCMA—directed therapy naive population. Changing targets after relapse from a BCMA—targeted therapy (e.g., switching to a GPRC5D—targeted therapy) appears to work well. Further studies, including biological studies, are needed to understand the expression of surface molecules, how they vary over time and at the time of relapse, how much they influence the clinical presentation of the disease, and to better identify the role of potential drug targets in myeloma, including the orphan receptor GPRC5D, together with robust efficacy and safety data from clinical trials and real-world experience: this will better help us to identify a valid and rational strategy for sequencing. To date, based on our analysis of the available literature data, GPRC5D appears to be a promising target for effective therapies, even in very advanced stages of the disease.

### Supplementary Information

Below is the link to the electronic supplementary material.

Supplementary file1 (DOCX 14 KB)
