# Supplementary material for: Beyond BCMA, why GPRC5D could be the right way: treatment strategies with immunotherapy at relapse after anti-BCMA agents
Source: Cancer Immunol Immunother. 2023 Nov 4;72(12):3931–7. doi: 10.1007/s00262-023-03559-4 (PMC10700430; doi:10.1007/s00262-023-03559-4)
Supplement: Supplementary file 1 — Supplementary file1 (DOCX 14 KB) [file 262_2023_3559_MOESM1_ESM.docx]

**Table 3**. Anti-BCMA exposed patients’ outcome in studies evaluating G protein-coupled receptor class C group 5 member D (GPRC5D)-targeted chimeric antigen receptor (CAR) T cell therapy

| References | no. pts. anti-BCMA exposed (%) |  | no. pts with PR or better | ORR % |
| --- | --- | --- | --- | --- |
| Mailankody, 2022^51^ | 10 (59) |  | 7 | 70 |
| Bal, 2022^52^ | 9 (27) |  | 9 | 100 |
| Huang, 2022^53^ | 5 (50%) |  | 5 | 100 |

BCMA, B cell maturation antigen; no., number; ORR, overall response rate; pts, patients.
